# Deep Learning-Based Detection of Pigment Signs for Analysis and Diagnosis of Retinitis Pigmentosa

**DOI:** 10.3390/s20123454

**Published:** 2020-06-18

**Authors:** Muhammad Arsalan, Na Rae Baek, Muhammad Owais, Tahir Mahmood, Kang Ryoung Park

**Affiliations:** Division of Electronics and Electrical Engineering, Dongguk University, 30 Pildong-ro 1-gil, Jung-gu, Seoul 04620, Korea; arsal@dongguk.edu (M.A.); naris27@dongguk.edu (N.R.B.); malikowais266@gmail.com (M.O.); tahirmahmood.cs@gmail.com (T.M.)

**Keywords:** deep learning, retinal disease, retinitis pigmentosa, semantic segmentation, RPS-Net

## Abstract

Ophthalmological analysis plays a vital role in the diagnosis of various eye diseases, such as glaucoma, retinitis pigmentosa (RP), and diabetic and hypertensive retinopathy. RP is a genetic retinal disorder that leads to progressive vision degeneration and initially causes night blindness. Currently, the most commonly applied method for diagnosing retinal diseases is optical coherence tomography (OCT)-based disease analysis. In contrast, fundus imaging-based disease diagnosis is considered a low-cost diagnostic solution for retinal diseases. This study focuses on the detection of RP from the fundus image, which is a crucial task because of the low quality of fundus images and non-cooperative image acquisition conditions. Automatic detection of pigment signs in fundus images can help ophthalmologists and medical practitioners in diagnosing and analyzing RP disorders. To accurately segment pigment signs for diagnostic purposes, we present an automatic RP segmentation network (RPS-Net), which is a specifically designed deep learning-based semantic segmentation network to accurately detect and segment the pigment signs with fewer trainable parameters. Compared with the conventional deep learning methods, the proposed method applies a feature enhancement policy through multiple dense connections between the convolutional layers, which enables the network to discriminate between normal and diseased eyes, and accurately segment the diseased area from the background. Because pigment spots can be very small and consist of very few pixels, the RPS-Net provides fine segmentation, even in the case of degraded images, by importing high-frequency information from the preceding layers through concatenation inside and outside the encoder-decoder. To evaluate the proposed RPS-Net, experiments were performed based on 4-fold cross-validation using the publicly available Retinal Images for Pigment Signs (RIPS) dataset for detection and segmentation of retinal pigments. Experimental results show that RPS-Net achieved superior segmentation performance for RP diagnosis, compared with the state-of-the-art methods.

## 1. Introduction

Retina is among the highest metabolically active tissues in the body, and different diseases can cause structural changes in the retina. These changes can be identified for diagnostic purposes. Retinal imaging by optical coherence tomography (OCT) and fundus imaging can help in the analysis of eye diseases. These diseases include diabetic retinopathy, macular degeneration, retinitis pigmentosa (RP), macular edema, macular bunker, and glaucoma [1]. Among these diseases, RP is a rare eye disease with a prevalence of 1/4000 which is caused by degeneration of the cones and rods by a gene mutation. An early clinical feature of RP is night blindness, which is later converted to the loss of peripheral vision and finally can lead to complete blindness [2]. A retinal image of RP shows pigmented areas on the retina posterior which can increase and migrate with symptoms. The RP state can be analyzed using OCT and fundus imaging [3]. RP diagnosis and growth rate determination are very important for early treatment. Studies have focused on the growth rate of RP for stem cell therapy and surgery for RP [4]. Monitoring RP progress through retinal imaging morphology and genotyping could benefit gene therapy [5]. Learning-based methods using deep/machine learning have attracted attention for the screening of various retinal diseases and for abnormality detection for disease analysis. These diseases can be identified through classification, or can be segmented for deep analysis [6]. Glaucoma and related diseases can be classified using deep learning methods that can discriminate between normal and diseased eyes from large amounts of data [7]. Considering the importance of segmentation of retinal diseases, many researchers have focused on retinal vessels, optical disc, optical cup, optic nerve, and disease spot segmentation for analysis of different diseases [8,9,10,11,12,13,14]. Clinically advanced solutions have been proposed to detect diabetic and hypertensive retinopathy by deep learning-based segmentation [15]. Considering RP, medical researchers are focusing on the classification of RP severity using scoring criteria based on visual acuity, visual field width, and ellipsoid zone width. A score between 0–15 describes the severity of the RP based on the results of imaging findings [16]. Researchers are currently focusing on developing low-cost fundus imaging solutions for retinal disease identification and analysis. These solutions can then be implemented on mobile platforms for clinical purposes [17]. Artificial intelligence-based algorithms and semantic segmentation are already helping the healthcare sector in detection and diagnosis of various retinal and other diseases [18,19,20,21,22,23,24,25]. It is now possible to create a low-cost fundus image solution for the detection and segmentation of pigment signs for RP analysis. Very few studies have focused on RP detection using fundus images. To implement a robust system for RP detection and accurate segmentation of pigment signs (PS), we propose an effective deep learning-based RP segmentation network (RPS-Net). RPS-Net can accurately detect the PS deposited on the retina due to cone degeneration. In addition, RPS-Net accurately segments these pigments for analysis and to determine the growth rate of the disease. Unlike conventional deep learning-based segmentation networks, RPS-Net focuses on feature importation, which ensures the detection of PS even with few pixels. Our proposed RPS-Net is a fully convolutional network that avoids the use of fully connected layers, thus allowing the network to utilize less trainable parameters, and numerous dense paths enable the network to provide fine segmentation performance without prior preprocessing.

Unlike the other OCT-based RP detection algorithms, this study focuses on fundus imaging for RP analysis. The RPS-Net provides fine segmentation with inferior quality fundus images to aid the ophthalmologist or medical practitioner in the detection and analysis of growth in RP patients. Compared to available methods, this study is novel in the following three ways:

- RPS-Net avoids the postprocessing step to enhance segmentation results.
- RPS-Net utilizes deep-feature concatenation inside and outside of the encoder-decoder to enhance the quality of the feature.
- For fair comparison with other research results, the trained RPS-Net models and algorithms are made publicly available through [26].

The paper is structured as follows: Section 2 covers the related works. Section 3 describes the methodology of the proposed system and structure of the RPS-Net. Section 4 provides experimental visual results and evaluation. Section 5 elaborates the discussion on the clinical usage of the proposed method. Section 6 presents the conclusive remarks and the future directions of the proposed work.

## 2. Related Works

Segmentation of retinal images is a well-known method for diagnosis and analysis of several diseases. Specifically, with respect to RP, few researchers have focused on segmentation of fundus images. A low-cost solution based on a learning-based method can produce good results with few images. Researchers have focused on different spot segmentation techniques of retinal images. The retinal image segmentation of fundus images is explained using two major groups of methods: handcrafted-feature and deep-feature-based methods.

### 2.1. Retinal Image Segmentation Based on Handcrafted Features

Considering the detection of retinal disease landmarks, Sánchez et al. proposed a novel method for hard exudate segmentation based on color information using Fisher’s linear discriminant model with prior preprocessing of the image [27]. Zhang et al. presented a specific preprocessing method that performs normalization and denoising, where mathematical morphology is used for exudate detection [28]. Welfer et al. presented a coarse-to-fine strategy for detection of the hard exudates based on mathematical morphology, thresholding, and maximal transform [29]. Considering the retinal pigment epithelium, Götzinger et al. used intensity-based segmentation based on polarization; this scheme is implemented on polarization-sensitive optical coherence tomography (PS-OCT) [30]. Yang et al. used dual-scale gradient information combined with a Canny edge detector for the inner and outer segments (IS/OS) in OCT images of RP patients [31]. This study focuses on the detection of RP in fundus images, which is underrepresented by previous studies. Das et al. [32] considered the fundus images for RP analysis using handcrafted local features. They extracted low and high-intensity levels from red and green channels, and the final images with microaneurysms and RP signs were segmented using a Sobel operator and interpolation of the original image. Considering the retinal pigment sign segmentation, Ravichandran et al. utilized the RIPS dataset. The mean filter was used for shade correction, and the contrast limited adaptive histogram equalization (CLACHE) was applied to enhance the image, where the watershed transform was used to detect the pigment deposits on the fundus images [33].

### 2.2. Retinal Image Segmentation Based on Deep-Feature (CNN)

Among the deep learning methods for retinal disease segmentation, Guo et al. used deep learning-based methods of DeepLab v2 and a fully convolutional residual network in a combination of bin loss function for hard exudate segmentation [34]. Mo et al. considered the cascaded residual network for exudate segmentation to recognize diabetic macular edema [35]. Similarly, the exudate landmarks were segmented by Prentašić et al. using the convolutional neural network; to segment other structures, the outputs of vessels and the optic disc were combined with exudates [36]. Tan et al. proposed a 10-layered convolutional neural network which could automatically detect the exudates in a multiclass manner for hemorrhages and microaneurysms in the retinal images. Image normalization was used before training and testing [37]. Many disease classification and detection methods related to the lesion, vessels, optical disc, and optical cup based on artificial intelligence were discussed by [38]. Chudzik et al. presented a fully convolutional deep learning method for microaneurysm detection. They used the patch-based classification, in which the required patches were generated after preprocessing with the green channel [39]. Phasuk et al. proposed an automatic glaucoma screening method, the approach used several classification networks and the output of those networks are combined to provide a simple artificial neural network (ANN) to provide the final prediction for the screening of the disease [40]. Christopher et al. used a deep neural network approach to predict the glaucomatous visual field damage in the OCT images, for this purpose they used ResNet-50 architecture with pretrained weights from ImageNet, where preprocessing is also used prior to the training of the network [41]. Martin et al. detected glaucoma eye diseases by means of computer aided diagnosis, they combined several networks trained for classification and segmentation tasks for glaucoma and these relevant structures and morphological features are combined to interpret the glaucoma disease on mobile platforms [42]. Fu et al. segmented the optical disc and optical cup to compute the cup to disc ratio (CDR) which is used to screen for glaucoma disease. In detail, they used a U-shaped network for the joint segmentation of OC and OD in a multiclass scenario with the help of multi-label loss functions [43]. Wang et al. also presented a glaucoma screening method using OC and OD segmentation by adversarial learning. Therefore, they used special domain adaption to generate the smooth segmentation and further developed the patch-based fine-grained discrimination on local segmentation details for effective performance [44]. The more detailed eye disease detection in fundus images are effectively discussed by Islam et al. [45]. As described in Section 2.1, most researchers have focused on OCT images for RP. Because of the unavailability of the public dataset for RP and pigment landmarks, few researchers have focused on learning-based methods for retinal pigment segmentation with fundus images for RP analysis. Brancati et al. [46] innovatively constructed the Retinal Images for Pigment Signs (RIPS) dataset for segmentation of retinal pigments and detection of RP in fundus images. They provided baseline learning-based methods for the researchers, which contributed to RP analysis. They used a three step method to detect the pigment signs. In the first step, preprocessing is applied to correct the lighting and noise effects, illumination correction is carried out by shade correction method, and high frequencies are handled edge preserving smoothing. In the second step, watershed transform is used to divide the image into homogenous components after changing the preprocessed image into Lab color space, where the number of regions is limited by the Otsu multi-level method and the region merging process is performed. In the third step, AdaBoost and Random Forest classifiers are used to classify the selected regions in the previous steps. The strength of this method is the simplicity of the ensemble learning and the advantage over the classification trees. As false negatives are more important in medical applications, so the Random Forest classifier shows more false negatives compared to AdaBoost [46]. The same group subsequently enhanced the accuracy further by using a modified U-Net deep learning model on patches [47]. Therefore, the two blocks removed from the original five U-Net blocks and the number of filters is halved. Avoiding the preprocessing schemes and a substantial increase in F-measure compared to the machine learning method are the strength of this method. Considering the F-measure, this method improved overall segmentation performance, but as false negatives (represented by sensitivity) are considered more critical than false positive, this method [47] has higher false negatives (low sensitivity) compared to Random Forest and AdaBoost presented in [46].

Table 1 shows the strengths and weaknesses of the retinal pigment segmentation in contrast with RPS-Net for RP analysis.

## 3. Proposed Method

### 3.1. Overview of the Proposed Architecture

Unlike image classification networks, RPS-Net is a fully convolutional network that does not include a fully connected layer. RPS-Net provides accurate pixel-wise classification and marks the detected pixel using a pixel classification layer. Because the proposed method takes advantage of deep-feature concatenation for both the encoder and decoder, the network can import and concatenate high-frequency information from different layers. Because of multiple dense connections, RPS-Net is powerful in the segmentation of PS in intense scenarios. The RPS-Net takes the original fundus image as direct input into the RPS-Net without any preprocessing, and it gives the retinal pigment mask, which is detected as output without postprocessing.

### 3.2. Retinal Pigment Sign Segmentation Using RPS-Net

**Figure 1:**
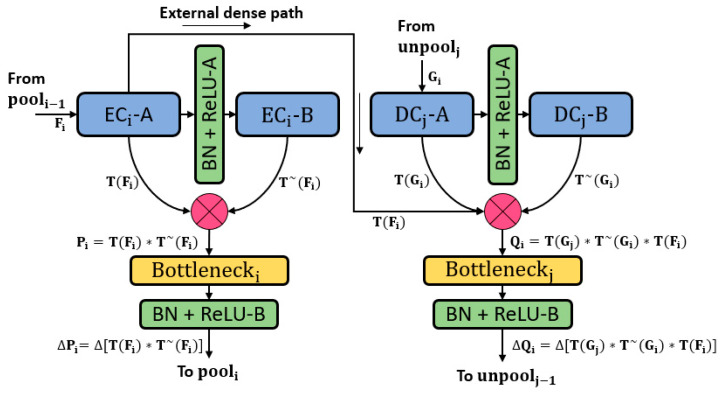
Schematic diagram of retinitis pigmentosa segmentation network (RPS-Net) deep-feature concatenation.

The classification task is the basis for computer vision tasks like detection, segmentation, et cetera. To accomplish the classification task, the neural networks have to become deeper with many convolutional layers. These convolutional layers tend to lose spatial information in each operation, which is logically called the vanishing gradient problem [48]. The most well-known approach to deal with the vanishing gradient problem is feature empowerment using ResNet [49], which when applied skips connections based on summation, thereby creating a valuable performance increment. However, the residual networks can still face the information flow impedance problem, which can be alleviated using DenseNet, which provides dense connectivity by deep-feature concatenation [50]. DenseNet outperforms the well-known networks for classification because of the reduced number of parameters [50]. RPS-Net adopts a similar deep-feature concatenation method using dense connections. As the retinal pigments are very small, dealing with classes with a low number of pixels is a difficult task. The segmentation task is accomplished on the basis of three techniques. First, feature loss by convolution should be compensated within the dense block by deep-feature concatenation. Second, these immediate enriched features should be transferred from the encoder to the decoder by the same deep-feature concatenation. Third, because convolutions cause loss of information in a combination of pooling operations in each block, the number of convolutions should be lower than those of conventional networks. Figure 1 represents the deep-feature concatenation layout for the candidate encoder-decoder block. The three strategies discussed above are implemented in the design of RPS-Net which let it perform segmentation with just 10.5 million trainable parameters.

**Figure 2:**
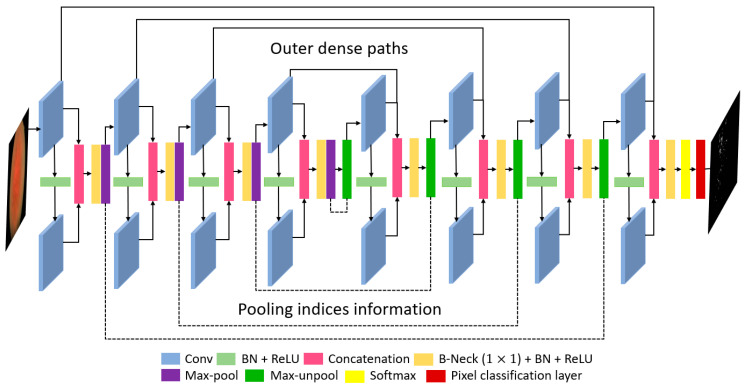
Proposed RPS-Net architecture for retinal pigment segmentation.

The quality of the retinal images taken from the fundus camera is usually compromised because of image acquisition conditions. Figure 2 represents the complete architecture with dense feature implementation policy by concatenation. Each encoder and each decoder comprise four dense blocks with two convolutional layers in each block. As shown in Figure 1 which shows the candidate dense block, each encoder block (left of Figure 1) receives an input feature $F_{I}$ and each decoder block receives an input feature $G_{i}$. The convolutional operation from the first convolution of the encoder ${\mathrm{EC}}_{i}$-A gives the resultant feature $T(F_{i})$, which is provided to the second convolution of the encoder ${\mathrm{EC}}_{i}$-B which alters the feature to $T^{\sim }(F_{i})$. The feature $T^{\sim }(F_{i})$ is obtained after two convolutional operations, and the spatial loss is recovered by deep-feature concatenation of these two convolutional layers. The dense feature $P_{i}$ and the concatenated feature of the outputs ($T(F_{i}){\;\mathrm{and}\;T}^{\sim }(F_{i})$) of ${\mathrm{EC}}_{i}$-A and ${\mathrm{EC}}_{i}$-B are given as follows:

(1) $$P_{i}=T(F_{i}){*T}^{\sim }(F_{i})$$

Here, $P_{i}$ is the dense feature after concatenation of $T(F_{i}){\;\mathrm{and}\;T}^{\sim }(F_{i})$, where “*” represents the depth-wise concatenation. The number of channels for the $P_{i}$ feature is increased, which can cause memory consumption. Therefore, ${\mathrm{Bottleneck}}_{i}$ limits the channels after the batch normalization and ReLU operation. The controlled feature is $\Delta P_{i}$, which is given by the following equation:

(2) $$\Delta P_{i}=\Delta [T(F_{i}){*T}^{\sim }(F_{i})]$$

Here, “Δ“ represents the batch normalization and ReLU operation in a combination of channel limitation by the ${\mathrm{Bottleneck}}_{I}$ layer, and ”*“ represents the depth-wise concatenation. Similarly, for the decoder (right of Figure 1), the convolutional operation from the first convolution of the decoder ${\mathrm{DC}}_{j}$-A gives the resultant feature $T(G_{i})$, which is provided to the second convolution of the decoder ${\mathrm{DC}}_{j}$-B, which alters the feature to $T^{\sim }(G_{i})$. The feature $T^{\sim }(G_{i})$ is obtained after two convolutional operations, and the spatial loss is recovered by deep-feature concatenation of these two convolutional layers. In addition, the third feature $T(F_{i})$ is imported from the encoder by the external dense path. Therefore, the dense feature $Q_{i}$ is an enriched feature by concatenation of three features $T(G_{i}),{\;T}^{\sim }(G_{i}),\;\mathrm{and}\;T(F_{i})$ of the outputs ${\mathrm{DC}}_{i}$-A, ${\mathrm{DC}}_{i}$-B, and ${\mathrm{EC}}_{i}$-A, which are given as follows:

(3) $$Q_{i}=T(G_{j}){*T}^{\sim }(G_{i})*T(F_{i})$$

Here $Q_{i}$ is the dense feature after concatenation of three features $T(G_{j}),{\;T}^{\sim }(G_{i})$, and $T(F_{i})$, where “*” represents the depth-wise concatenation. The number of channels for the $Q_{i}$ feature are increased, which can cause memory consumption. Therefore, ${\mathrm{Bottleneck}}_{I}$ limits the channels after batch normalization and ReLU operation. The controlled feature is $\Delta Q_{i}$, given by the following equation:

(4) $$\Delta Q_{i}=\Delta [T(G_{j}){*T}^{\sim }(G_{i})*T(F_{i})]$$

Here, “Δ” represents the batch normalization and ReLU operation in a combination of channel limitation by the ${\mathrm{Bottleneck}}_{j}$ layer, and ”*“ represents the depth-wise concatenation. Both $\Delta P_{i}$ and $\Delta Q_{i}$ features are empowered by dense connectivity. However, $\Delta Q_{i}$ is a resultant feature of concatenation of three features, which include the important edge information enriched feature $T(F_{i})$ which lets the RPS-Net perform fine segmentation without prior preprocessing.

**Figure 3:**
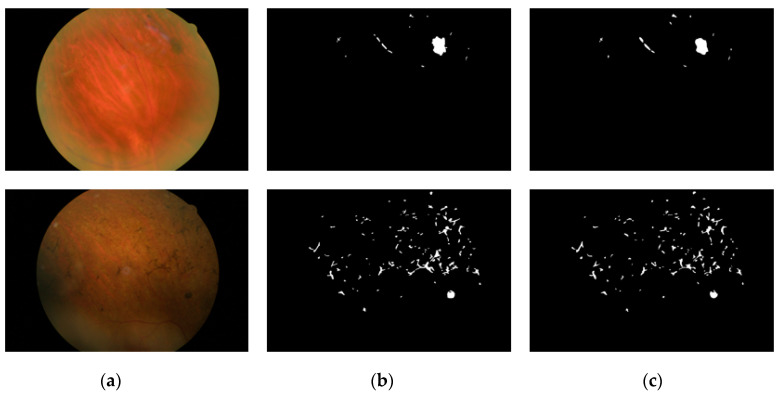
Sample RP Fundus images with and ground-truths for the RIPS dataset: (**a**) original images, (**b**) G1, and (**c**) G2.

There are three design concerns for RPS-Net: First, to ensure the segmentation of the small object dense block level, feature enhancement is performed, which is shown for each dense block in Figure 3 (encoder side) and represented by $P_{i}$ in Figure 1; Second, importation of immediate features from the initial dense block to decoder enables further enhancement before pixel classification, which is shown in Figure 2 (decoder side) and represented by $Q_{i}$ in Figure 1; Third, the overall reduction of the convolutional layers and pooling layers is implemented using four dense blocks for each encoder and decoder, respectively. The RPS-Net maintains the feature map size before upsampling at 18 × 25 for an input image of 400 × 300, which is sufficient to represent the valuable features for retinal pigment segmentation.

Considering the key architectural differences from semantic segmentation architectures of SegNet [51], OR-Skip-Net [52], Vess-Net [15], U-Net [53], Modified U-Net [46], Dense-U-Net [54], H-Dense-U-Net [54], and U-Net++ [55]. The RPS-Net follows the very simple architecture of two convolutional layers in each dense block (for both encoder and decoder). The features from these two convolutional layers in each dense block are concatenated locally inside that specific block (connectivity-1). Additionally, from each first convolutional layer the feature of each encoder block is directly provided to the corresponding decoder convolution (connectivity-2) as shown in Figure 2. The local feature concatenation (connectivity-1) combines two inputs, where outer feature concatenation (connectivity-2) combines three inputs. Table 2 presents the potential architectural differences for the proposed RPS-Net in contrast to existing convolutional neural networks such as, SegNet [51], OR-Skip-Net [52], Vess-Net [15], U-Net [53], Modified U-Net [46], Dense-U-Net [54], H-Dense-U-Net [54], and U-Net++ [55]. Note that these mentioned networks are used in different domains of computer vision applications.

The RPS-Net encoder performs a constant convolutional operation on the image, and the feature travels through the network in a feed-forward fashion until the image is represented by small features. The convolution and max-pooling operation cause spatial information loss, because of which useful information can be lost with the other information. This is avoided by deep-feature concatenation in RPS-Net. By the four dense block operations with eight convolution layers and four pooling layers, the final feature map is 18 × 25 for a 400 × 300 input image. Note that, the RPS-Net is designed with the feature reuse policy phenomena, according to which it receives an input image of 400 × 300 × 3 which is a color image. The structure of the RPS-Net encoder in terms of the dense block is shown in Table 3, the max-pooling operation reduces the feature map size, which accelerates the network computation. The pooling layers in RPS-Net have information of the indices, as shown in Figure 2. The paths of these information indices do not contain the image but contain the image size and index information, which is transferred to the corresponding unpooling layer at the decoder side

As shown in Figure 2, RPS-Net decoder provides the reverse operation of the decoder. Unlike the encoder, each dense block in the decoder starts with an unpooling layer which increases the feature map size gradually using the pooling indices from the encoder. After each unpooling layer, the encoder follows the same process for the connectivity of the convolutional layers in the decoder. The feature maps of both convolutional layers are concatenated by a depth-wise concatenation layer. The RPS-Net decoder receives an input of 18 × 25 pixels from the encoder and provides the final feature map of the size equal to the input image provided to the network. The purpose of the RPS-Net is to perform semantic segmentation on retinal images to provide a pixel-wise classification for RP. The pixel classification layer in combination with softmax is responsible for assigning a label to each pixel in the image from the available class based on prediction. Table 4 provides the layer layout of the RPS-Net decoder with the respective feature map sizes.

## 4. Experimental Results

### 4.1. Experimental Data and Environment

This study uses retinal images for the detection of retinal PS. Because this study is based on the rare retinitis pigmentosa disease with applications for the method of RP analysis to aid the medical practitioner in early diagnosis of the disease, we used RIPS dataset which is the only publicly available real dataset [46]. The same RIPS dataset was solely used by previous studies [33,46,47]. Moreover, to validate the performance of RPS-Net, 4-fold cross-validation is used with different patients for training and testing. In details, the RIPS dataset consists of images from four different patients captured using a Canon CR4-45NM retinal camera; the data of each patient are called one fold. Each fold contains 30 images (of 1440 × 2160 pixels), resulting in a total of 120 images for four patients. Therefore, for each patient, five images each of the left and right eye are taken in three different sessions, which creates 30 images for each patient (5 images × 3 sessions = 15 images for each eye). The period between two consecutive sessions varied from one to six months, where the total period between first and last sessions always exceeded one year. Of the 120 images, 99 images were of RP (retinal pigments), whereas 21 images were of healthy eyes. Two ophthalmologists provided separate manual segmentation masks (G1 and G2) for PS, where the further details for manual mask generation can be found in [46]. Figure 3 shows an example of a retinal image and the corresponding ground truth (G1 and G2) mask from the RIPS.

In this study, to reduce memory usage during training and testing, the images and labels were resized to 300 × 400 pixels for both original images and the ground-truth images. To train the RPS-Net, from the total of four folds, three folds were used for training and the remaining one fold was applied for testing with 4-fold cross-validation criteria similar to that used by [46]. Data augmentation was applied to artificially increase the amount of training data, to ensure better training. The procedure of data augmentation is explained in Section 4.2.

The RPS-Net was trained and tested using a desktop computer with Intel Core i7-3770K CPU with 3.50 GHz clock speed (4 cores), 28 GB RAM, and an NVIDIA GeForce GTX Titan X graphical processing unit (GPU with 12 GB of graphics memory and 3072 CUDA cores) [56]. In this study, the RPS-Net experiments were performed from scratch using MATLAB 2019b [57]. Note that the RPS-Net was trained with our training dataset, which did not undergo fine-tuning or weight initialization from other networks.

### 4.2. Data Augmentation

**Figure 4:**
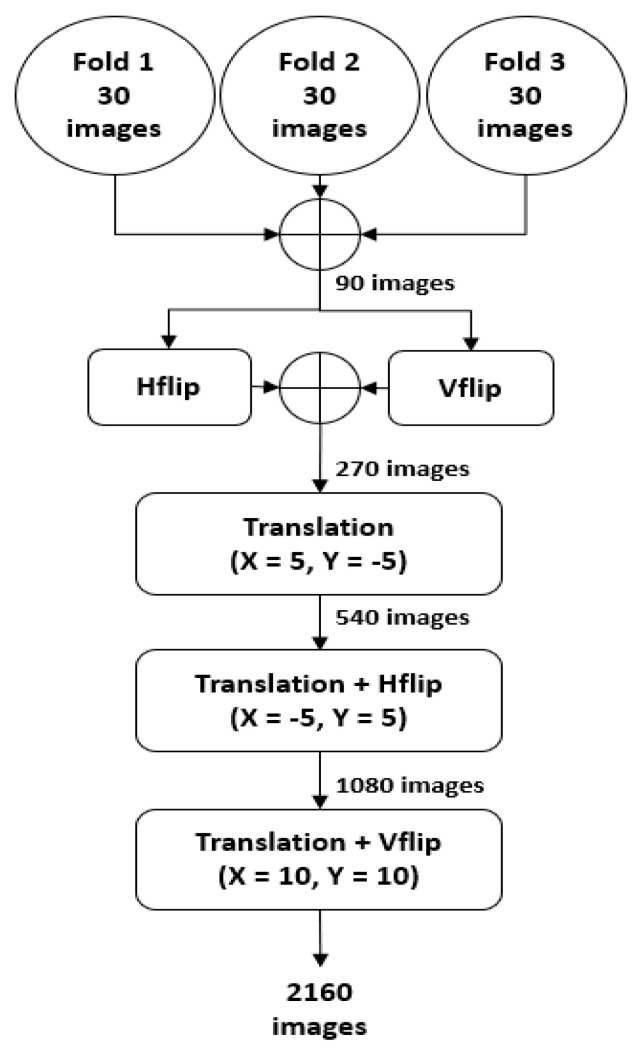
Illustration of the data augmentation process used to generate artificial images to train RPS-Net; Hflip and Vflip represent the horizontal flip and vertical flip, respectively.

As mentioned in Section 4.1, the RPS-Net was trained with three-fold images and tested with the fourth fold of different patients using 4-fold cross-validation. The three folds consist of 90 images, which are not sufficient to train the RPS-Net. Therefore, artificial images were generated using the training images (90 for each fold) through the data augmentation process. In detail, the three folds were combined to make 90 images, and these images were horizontally and vertically flipped (H-flip and V-flip) to create 90 images each, which made a total of 270 images (90 (three folds)+ 90 (H-flip) + 90 (V-flip) = 270). These 270 images are then XY translated (X = 5, Y = −5) to make 540 images. In the next step, these 540 images were then XY translated (X = −5, Y = 5) again with a horizontal flip to create 1080 images. In the final step, the 1080 images from the previous stage were then XY translated (X = 10, Y = 10) with a vertical flip to create a total of 2160 images. A detailed visualization of the augmentation process is represented in Figure 4.

### 4.3. RPS-Net Training

RPS-Net focuses on immediate information transfer between layers by dense connectivity. Each dense block in the encoder-decoder block provides dense connectivity. This type of connectivity helps the network to converge with rich features to detect PS. The RPS-Net was trained using augmented data of three folds (explained in Section 4.2). The RPS-Net backbone (encoder-decoder) was designed by us and trained from scratch without any weight sharing or initialization from other networks. To ensure that the benefits of the Adam optimizer [58] are retained over those of conventional stochastic gradient descent, Adam was chosen as the optimizer which has a learning rate of 0.0001. The RPS-Net is trained for 20 epochs with 43,200 iterations with a minibatch size of 10 images per iteration. To provide variant features during training, the images were shuffled in each epoch. The mentioned learning rate was kept constant during the training with an epsilon value of 0.000001 with global L2 normalization, which is smooth and rotationally invariant. As shown in Figure 3c,d, there is a considerable difference between pixel numbers of both classes (“pigment” and “background”). The PS has very few pixels, whereas the backgrounds have a large number of pixels. To maintain fast network converge, the weight balancing was used by median frequency balancing. Further details of frequency balancing can be found in [51,52].

### 4.4. Testing of the Proposed Method

#### 4.4.1. RPS-Net Testing for Pigment Sign Segmentation

RPS-Net is based on dense block-level feature concatenation to internally empower the feature maps. In contrast, the RPS-Net encoder provides the outer dense paths, which provide immediate information directly to the corresponding layers, and allows the network to learn rich information. RPS-Net is a densely powered semantic segmentation network that does not require prior image preprocessing to detect retinal pigments. The RPS-Net takes an image of 300 × 400 × 3 pixels as input directly and performs continuous convolutional operations to recognize the pigment spots in the retinal images in a feed-forward fashion. In detail, RPS-Net is based on eight local dense connections for the encoder and decoder (4 each), which connect both convolutional layers of each block densely. In addition, there are four outer dense connections which densely connect each first convolutional layer of each block with corresponding layers directly in the decoder. At the output, RPS-Net provides two predicted binary masks for each “pigment” and ”background” class based on trained knowledge. Furthermore [46], RPS-Net was evaluated based on sensitivity (Sen), specificity (Spe), precision (P), accuracy (Acc), and F-score (F) which are given by the following equations:

(5) $$\mathrm{Sen}=\frac{TP}{TP+FN}$$

(6) $$\mathrm{Spe}=\frac{TN}{TN+FP}$$

(7) $$P=\frac{TP}{TP+FP}$$

(8) $$\mathrm{Acc}=\frac{TP+TN\;}{TP+TN+FP+FN}$$

(9) $$F-\mathrm{score}=\frac{2TP\;}{2TP+FP+FN}$$

where *TP*, *FN*, *FP*, and *TN* are the number of true positive, false negative, false positive, and true negative pixels, respectively. *TP* is the pixel that is listed as the “Pigment” pixel in the ground-truth image and is predicted as “Pigment” by our method. *FN* is the pixel listed as “pigment” in the ground-truth image and predicted as “background” by our method. *FP* is the pixel listed as “background” in the ground-truth image and is predicted as “pigment” by our method. *TN* is the pixel listed as “background” in the ground-truth image and is correctly predicted as “background” by our method.

#### 4.4.2. Retinal Pigment Sign Segmentation Results by RPS-Net

**Figure 5:**
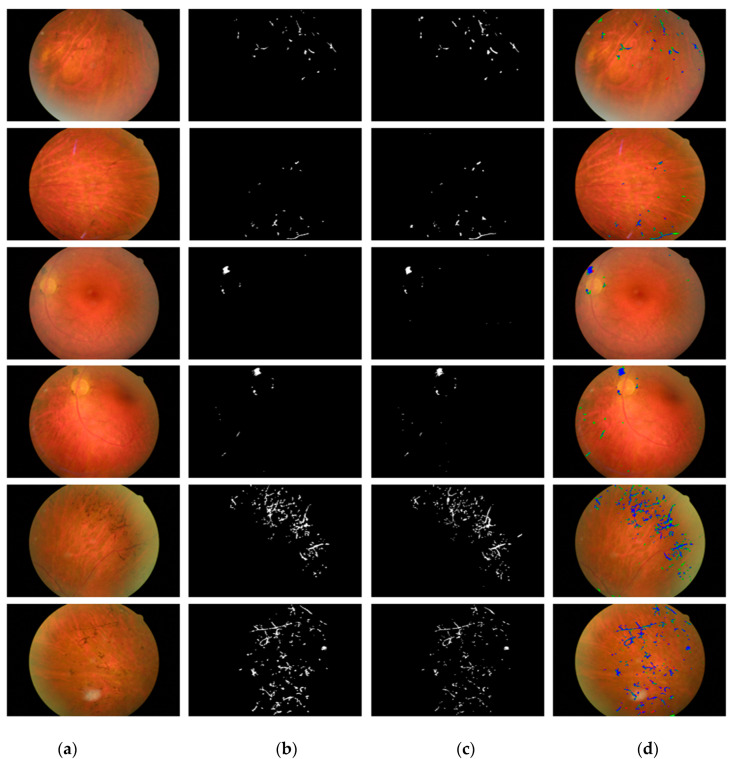
Examples of RPS-Net results for pigment sign segmentation for the Retinal Images for Pigment Signs (RIPS) dataset: (**a**) original retinal image; (**b**) ground-truth mask G1; (**c**) ground-truth mask G2; (**d**) predicted retinal pigment mask by RPS-Net, where *FP* is indicated in green, *FN* in red, and *TP* in blue.

**Figure 6:**
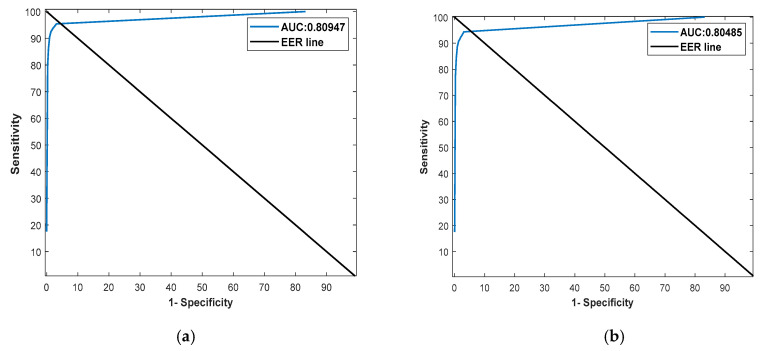
ROC curves for RPS-Net based on the ground-truth masks by (**a**) the first expert G1 and (**b**) the second expert G2.

The visual results for the retinal pigment segmentation by RPS-Net are shown in Figure 5, which follows the standard of *FP* (indicated in green), *FN* (indicated in red), and *TP* (indicated in blue). Figure 6a,b represents the ROC curve for the proposed method based on G1 and G2 of Table 5 and Table 6, respectively. They represent the relation between sensitivity and 1-Specificity. Therefore, the black straight line is the equal error rate (EER) line, and the intersection position of the EER line to the ROC curve represents that where sensitivity is equal to specificity. In Table 5 and Table 6, we compared the accuracies by our method with those by previous methods based on their reported accuracies because we followed the same experimental protocol with the same dataset and their algorithms are not open. Therefore, we cannot draw the ROC curves of the previous methods, and include only the curves of our method. The ROC curves by the proposed RPS-Net represents the area under curve (AUC) of 0.80947 and 0.80485 for G1 and G2, respectively.

#### 4.4.3. Comparison of RPS-Net with Other Methods

In this section, the performance of RPS-Net is compared with those of existing methods based on sensitivity (Sen), specificity (Spe), precision (P), accuracy (Acc), and F-score (F), as shown in Section 4.4.1 using Equations (5)–(9). Considering the original image size of 1440 × 2160, it is very difficult to train the network due to the limitation of GPU memory. Therefore, the images were resized to 400 × 300 size for the training and testing of the RPS-Net. However, we resized the segmented image on our network to 1440 × 2160 pixels by bi-linear interpolation to compare it with the original G1 and G2 of size 1440 × 2160 pixels and made fair comparisons with [33,46,47]. Table 5 presents the numerical results for retinal pigment sign detection based on G1, whereas Table 6 presents the same results based on G2. As shown in Table 5, most accuracies including Sen, Spe, F, and Acc by our method are higher than those by the state-of-the-art methods although P by our method is a little lower than that of the previous method [47]. In addition, our method outperforms the previous method [46] as shown in Table 6, and receiver operating characteristic (ROC) curves by our method are shown in Figure 6.

False negatives are much more serious than false positives, and the false negative pixels are judged by the sensitivity given by Equation (5). According to Table 5 and Table 6, the RPS-Net has high sensitivity, which represents the low number of false negative pixels.

## 5. Discussion

The detection of RP is based on the detection of PS. Medical practitioners analyze retinal images to note the progression of the disease [3]. These pigment migrations can be detected using the proposed method, which provides the option of computer-aided diagnosis to aid the ophthalmologist in the timely detection of RP. RPS-Net is based on dense feature empowerment which helps the network detect smaller pigment spots. The possible clinical outcomes from the proposed method are explained briefly in Section 4.1 and Section 4.2

### 5.1. Detection/Counting and Size Analysis of Retinal Pigments

**Figure 7:**
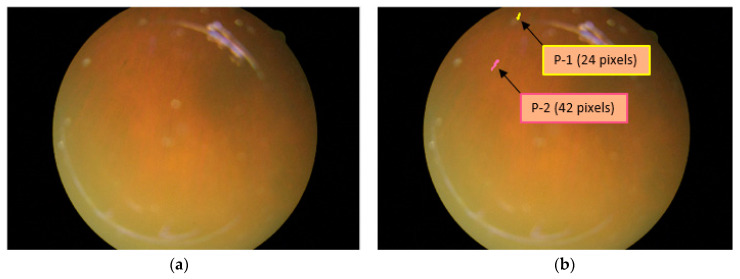
Sample image for retinal pigment sign detection, count, and size analysis. (**a**) Original image; (**b**) detected pigment spots with sizes.

The presence of RP can be detected with the segmentation of pigment spots, and if these pigment spots are detected over retinal images, they can be counted and analyzed for size by RPS-Net. Figure 7a shows an example of the original image in which the pigment signs are hardly visible. However, because of powerful dense connectivity, the RPS-Net detected two PS that are shown in yellow and pink in Figure 7b. Even the smaller PS were detected, and their sizes were found to be 24 and 48 pixels for the first (P-1) and second pigments (P-2), respectively. The sizes can be checked in consecutive visits of the patient to analyze disease progression.

### 5.2. Location Analysis for PS

**Figure 8:**
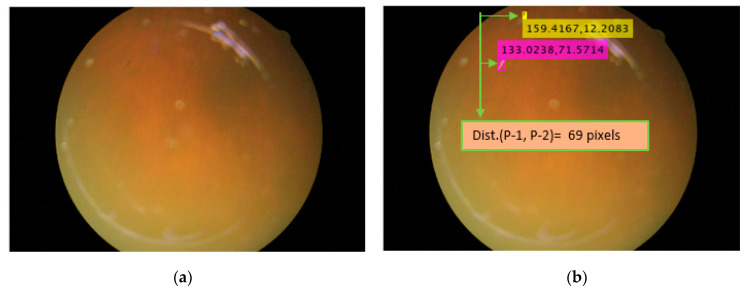
Sample image for retinal pigment sign detection, count, and size analysis. (**a**) Original image; (**b**) detected pigment spots with sizes.

The location of the retinal pigment is an important constraint for considering the progression and migration of spots [3,4]. In the retinal image, the X-Y location coordinates of the PS can be found for analysis of RP. For example, the X-Y coordinates of the pigments shown in yellow and pink are X = 159.4167 and Y = 12.2083 and X = 33.0238 and Y = 71.5714, respectively. The distance between the pigments observed between different visits of the patient can be another piece of information that can be useful for migration analysis of the PS. As shown in Figure 8a,b, the distance between the two spots can be found, which is approximately 69 pixels for this specific example.

## 6. Conclusions

In this study, we proposed RPS-Net, which is empowered by dense block-level deep-feature concatenation and external dense connections for immediate information transfer. The method can segment the retinal pigments with a low number of pixels. There are three important principles on which RPS-Net was based and designed: First, the dense block-level feature concatenation improves the quality of the features, and its dimensions are controlled by bottleneck layers to limit memory usage. Second, convolutions cause information loss; thus, to reduce the overall convolutions, only four blocks are used for each encoder and decoder. Third, conventional encoder-decoder-based networks do not pass edge information from the encoder to the decoder, which deteriorates feature maps in terms of edges and minor information. RPS-Net provides dense paths from the initial layers of the encoder to the decoder to fulfill the feature empowerment to segment minor level information. RPS-Net with immediate information flow inside and outside the encoder-decoder allows the network to converge quickly, in only 43,200 iterations. The proposed network provides good estimation of the size, location, counting, and distance information of the retinal pigments with correct segmentation for analysis of RP. This segmentation can assist medical practitioners or ophthalmologists to analyze the progression and intensity of the disease in a timely manner.

RPS-Net can detect and segment the retinal PS for diagnosis of RP. RPS-Net is a learning-based method, so robustness depends on the trained knowledge. The method can be used as a second-opinion system to aid doctors and ophthalmologists in the diagnosis and analysis of RP. In the future, we will enhance the accuracy of RP further. Using another low-cost network version, we will consider the diagnosis of other retinal diseases using artificial intelligence. Moreover, we intend to develop another method that can perform the segmentation with full image based on original image size.

## Figures and Tables

**Table 1:** Comparison of the available methods and RPS-Net for retinal pigment segmentation.

Type	Methods	Strength	Limitation
**RP by handcrafted features**	Das et al. [32]	Uses simple image processing schemes.	Preprocessing is required.
	Ravichandran et al. [33]	Watershed transform gives better region approximation.	The handcrafted feature-based method performance is subject to preprocessing by CLACHE.
**RP by learned features**	Brancati et al. [46]	The simple machine learning classifier are used, AdaBoost provides less false negatives	The classification accuracy of the classifier is based on the, denoising, shade correction, etc.
	Brancati et al. (Modified U-Net) [47]	Subsequently improved segmentation performance by modified U-Net model, and 15% improvement in F-measure compared to [46].	The method performance is affected by more false negatives (represented by sensitivity of the method) compared to [46].
	RPS-Net(Proposed)	Utilizes deep concatenation inside encoder-decoder, and encoder-to-decoder (outer) for immediate feature transfer and enhancement, with substantial reduction in false negatives.	Training for fully convolutional network requires a large amount of data by augmentation.

**Table 2:** Architectural differences between RPS-Net and existing deep learning models.

Method	Other Architectures	RPS-Net
SegNet [51]	Collectively, the network has 26 convolutional layers.	Total 16 convolutional layers (3 × 3) are used in the encoder and decoder with concatenation in each dense block.
	No feature reuse policy is employed.	Dense connectivity in both the encoder and decoder for feature empowerment.
	First two dense blocks have two convolutional layers, whereas the others have three convolutional layers.	Each dense block similarly has two convolutional layers.
	The convolutional layer with 512-depth is utilized twice in the network.	The convolutional layer with 512-depth is used once for each encoder and decoder.
OR-Skip-Net [52]	No feature reused policy is implemented for internal convolutional blocks.	Internal dense connectivity for both encoder and decoder.
	Only external residual skip paths are used.	Both internal and external dense paths are used by concatenation.
	No bottleneck layers are used.	Bottleneck layers are employed in each dense block.
	Total of four residual connections are used in total.	Overall, 20 dense connections are used internally and externally.
Vess-Net [15]	Based on residual connectivity.	Based on dense connectivity.
	Feature empowerment from the first convolutional layer is missed and has no internal or external residual connection.	Each layer is densely connected.
	No bottleneck layer is used.	Bottleneck layers are employed in each dense block.
	Collectively, 10 residual paths.	Overall, 20 dense connections are used internally and externally.
U-Net [53]	Overall, 23 convolutional layers are employed.	Total 16 convolutional layers (3 × 3) are used in the encoder and decoder with concatenation in each dense block.
	Up convolutions are for the expansion part to upsample the features.	Up convolutions are not used.
	Based on residual and dense connectivity.	Based on dense connectivity.
	Convolution with 1024-depth is used between the encoder and decoder.	1024-depth convolutions are ignored to reduce the number of parameters.
	Cropping layer is employed for borders.	Cropping is not required; pooling indices keep the image size the same.
Modified U-Net [47]	Overall, 3 blocks are used in each encoder and decoder	Overall, 4 blocks are used in each encoder and decoder
	The up convolutions are used for upsampling	Unpooling layers are used to upsample
	The deep feature concatenation is just used encoder-to-decoder	Feature concatenation used inside both encoder/decoder and encoder-to-decoder
	The number of filters considered is 32 to 128	The number of filters considered is 64 to 512
Dense-U-Net [54]	Total of 4 dense blocks are used inside encoder with 6, 12, 36, 24 convolutional layers in each block respectively	Total 16 convolutional layers for overall network with occurrence of two convolutional layers in each block
	Average pooling used in each encoder block	Max pooling used in each encoder block
	Five up convolutions are used in decoder for upsampling	4 unpooling layers are used in decoder for upsampling
H-Dense-U-Net [54]	Combines 2-D Dene-U-Net and 3-D Dene-U-Net for voxel wise prediction	Used for pixel wise prediction
	Total 4 dense blocks are used inside encoder with 3, 4, 12, 8 3-D convolutional layers in combination of 2-D Dense-U-net fusion	Total of 16 2-D convolutional layers for overall network
	Designed for 3-D volumetric features	Designed for 2-D image features
	Utilizes 3-D average pooling layer in each 3-D dense block	Used 2-Maxpooling layer in each encoder dense block
U-Net++ [55]	The external dense path is with dense convolutional block	No convolutional layer is used in external dense path
	There is a pyramid type structure of dense convolutional blocks between the encoder and decoder	Direct flat dense paths are used
	Individual dense blocks in dense path also have own dense skip connections	No convolutions are used in dense skip path

**Table 3:** RPS-Net encoder with deep-feature concatenation, individual feature map size of each block (DB, EC, EDP, Ecat, and Pool indicate dense block, encoder convolution, external dense path, encoder concatenation, and pooling layer, respectively). The layer that contains “^^” denotes batch normalization, and ReLU layers are associated with this layer. The table is designed with an input image size of 300 × 400 × 3.

Block	Name/Size	Number of Filters	Output Feature Map Size (Width × Height × Number of Channels)
**Encoder** **DB-1**	EC1-A ^^/3 × 3 × 3 To decoder (EDP-1) and Ecat-1	64	300 × 400 × 64
	EC1-B/3 × 3 × 64 To Ecat-1	64	
	Ecat-1 (EC1-A * EC1-B)	-	300 × 400 × 128
	Bneck-1^^/1 × 1 × 64		300 × 400 × 64
	Pool-1	-	150 × 200 × 64
**Encoder** **DB-1**	EC2-A ^^/3 × 3 × 64 To decoder (EDP-2) and Ecat-2	128	150 × 200 × 128
	EC2-B/3 × 3 × 64 To Ecat-2	128	
	Ecat-2 (EC2-A * EC2-B)	-	150 × 200 × 256
	Bneck-2^^/1 × 1 × 128		150 × 200 × 128
	Pool-2	-	75 × 100 × 128
**Encoder** **DB-1**	EC3-A ^^/3 × 3 × 64 To decoder (EDP-3) and Ecat-3	256	75 × 100 × 256
	EC3-B/3 × 3 × 64 To Ecat-3	256	
	Ecat-3 (EC3-A * EC3-B)	-	75 × 100 × 512
	Bneck-3^^/1 × 1 × 256		75 × 100 × 256
	Pool-3	-	37 × 50 × 256
**Encoder** **DB-1**	EC4-A ^^/3 × 3 × 64 To decoder (EDP-4) and Ecat-4	512	37 × 50 × 512
	EC4-B/3 × 3 × 64 To Ecat-4	512	
	Ecat-4 (EC4-A * EC4-B)	-	37 × 50 × 1024
	Bneck-4^^/1 × 1 × 512		37 × 50 × 512
	Pool-4	-	18 × 25 × 512

**Table 4:** RPS-Net decoder with deep-feature concatenation, and individual feature map size of each block (DB, DC, EDP, Dcat, and Pool indicate dense block, decoder convolution, external dense path, decoder concatenation, and pooling layer, respectively). The layer that contains “^^” denotes batch normalization, and ReLU layers are associated with this layer. The table is designed with an input image size of 300 × 400 × 3.

Block	Name/Size	Number of Filters	Output Feature Map Size (Width × Height × Number of Channels)
**Decoder** **DB-4**	Unpool-4	-	37 × 50 × 512
	DC4-B ^^/3 × 3 × 512 To Dcat-4	512	
	DC4-A/3 × 3 × 512 To Dcat-4	256	37 × 50 × 256
	Dcat-4 (DC4-B * DC4-A * EC4-A)	-	37 × 50 × 1280
	Bneck-5^^/1 × 1 × 1280	256	37 × 50 × 256
**Decoder** **DB-3**	Unpool-3	-	75 × 100 × 256
	DC3-B ^^/3 × 3 × 256 To Dcat-3	256	
	DC3-A/3 × 3 × 256 To Dcat-3	128	75 × 100 × 128
	Dcat-3 (DC3-B * DC3-A * EC3-A)	-	75 × 100 × 640
	Bneck-6^^/1 × 1 × 640	128	75 × 100 × 128
**Decoder** **DB-2**	Unpool-2	-	150 × 200 × 128
	DC2-B ^^/3 × 3 × 128 To Dcat-2	128	
	DC2-A/3 × 3 × 128 To Dcat-2	64	150 × 200 × 64
	Dcat-2 (DC2-B * DC2-A * EC2-A)	-	150 × 200 × 320
	Bneck-7^^/1 × 1 × 320	64	150 × 200 × 64
**Unpool-1** **DB-1**	Unpool-1	-	300 × 400 × 64
	DC1-B ^^/3 × 3 × 64 To Dcat-1	64	
	DC1-A/3 × 3 × 64 To Dcat-1	2	300 × 400 × 2
Dcat-1 (DC1-B * DC1-A * EC1-A)		-	300 × 400 × 130
**Bneck-8^^/1 × 1 × 130**		2	300 × 400 × 2

**Table 5:** Accuracies of retinal pigment sign segmentation by RPS-Net for the RIPS dataset based on the ground-truth mask by the second expert G2 (unit: %).

Type	Method	Sen	Spe	P	F	Acc
Handcrafted local feature-based methods	* Ravichandran et al. [33]	72.0	97.0	-	62.0	96.0
Learned/deep-feature-based methods	Random Forest [46]	58.26	99.46	46.18	47.93	99.14
	AdaBoost M1 [46]	64.29	99.30	42.45	46.76	99.01
	U-Net 48 × 48 [47]	55.70	99.40	48.00	50.60	99.00
	U-Net 72 × 72 [47]	62.60	99.30	46.50	52.80	99.00
	U-Net 96 × 96 [47]	55.20	99.60	56.10	55.10	99.20
	RPS-Net (proposed method)	80.54	99.60	54.05	61.54	99.52

**Table 6:** Accuracies of retinal pigment sign segmentation by RPS-Net for the RIPS dataset based on the ground-truth mask by the second expert G2 (unit: %).

Type	Method	Sen	Spe	P	F	Acc
Learned/deep-feature-based methods	Random Forest [46]	56.20	99.48	50.49	49.29	99.11
	AdaBoost M1 [46]	61.76	99.33	46.29	48.30	98.99
	RPS-Net (proposed method)	78.09	99.62	56.84	62.62	99.51
